# Lynch Syndrome Germline Mutations in Breast Cancer: Next Generation Sequencing Case-Control Study of 1,263 Participants

**DOI:** 10.3389/fonc.2020.00666

**Published:** 2020-05-29

**Authors:** Aleksey G. Nikitin, Daria A. Chudakova, Rafael F. Enikeev, Dina Sakaeva, Maxim Druzhkov, Leyla H. Shigapova, Olga I. Brovkina, Elena I. Shagimardanova, Oleg A. Gusev, Marat G. Gordiev

**Affiliations:** ^1^Pulmonology Research Institute, Federal Medical-Biological Agency of Russia, Moscow, Russia; ^2^School of Biological Sciences, University of Auckland, Auckland, New Zealand; ^3^Maurice Wilkins Centre for Molecular Biodiscovery, Auckland, New Zealand; ^4^Tatarstan Cancer Centre, Kazan, Russia; ^5^Department of Pharmacology, Bashkir State Medical University, Ufa, Russia; ^6^Druzhkov Clinic Ltd., Kazan, Russia; ^7^Extreme Biology Lab, Scientific and Clinical Center for Precision and Regenerative Medicine, Institute of Fundamental Medicine and Biology, Kazan Federal University, Kazan, Russia; ^8^Federal Research and Clinical Centre, Federal Medical-Biological Agency of Russia, Moscow, Russia; ^9^Kazan (Volga Region) Federal University, Kazan, Russia; ^10^KFU-RIKEN Translational Genomics Unit, RIKEN Cluster for Science, Technology and Innovation Hub, RIKEN, Yokohama, Japan; ^11^National Bioservice, Saint Petersburg, Russia

**Keywords:** breast cancer, *EPCAM*, Lynch syndrome, *MLH1*, *MSH2*, *MSH6*, *PMS2*, Targeted Next-Generation Sequencing

## Abstract

Genome instability—the increased tendency of acquiring mutations in the genome and ability of a cell to tolerate high mutation burden—is one of the drivers of cancer. Genome instability results from many causes including defects in DNA repair systems. Previously, it has been shown that germline pathogenic mutations in DNA Mismatch Repair (MMR) pathway cause cancer-predisposing Lynch Syndrome. We proposed that Lynch Syndrome-related germline mutations (LS-mutations) are associated with breast cancer (BC). In this study, we performed Targeted Next-Generation Sequencing of MMR pathway genes *MLH1, MSH2, MSH6, EPCAM*, and *PMS2* in a cohort of 711 patients with hereditary BC, 60 patients with sporadic BC, and 492 healthy donors. Sixty-nine patients (9.7%) with hereditary BC harbored at least one germline mutation in the MMR pathway genes, of them 32 patients (4.5%) harbored mutations in MMR pathway genes which we define as pathogenic or likely pathogenic, and of them 26 patients (3.6%) did not have any pathogenic mutations in DDR pathway genes, compared to two mutations in MMR pathway genes (0.4%) detected in a group of 492 healthy donors [*p* = 0.00013, *OR* = 8.9 (CI 95% 2.2–78.4)]. Our study demonstrates that LS-mutations are present in patients with hereditary BC more frequently than in healthy donors, and that there is an association of hereditary BC and mutations c.1321G>A in *MLH1*, c.260C>G and c.2178G>C in *MSH2*, c.3217C>T in *MSH6*, c.1268C>G and c.86G>C in *PMS2* genes. This finding provides a rationale for including pathogenic LS-mutations into genetic counseling tests for patients with hereditary BC.

## Introduction

Genome instability is one of the key hallmarks of cancer (1). The stability of genome is maintained in a cell by many mechanisms including DNA repair. The repair of DNA single-base mismatch and insertion/deletion loops occurring during DNA replication is executed by the Mismatch Repair (MMR) pathway ensuring mutation avoidance and precision of DNA replication (2). The MMR pathway proteins also take part in other cellular processes, and the whole spectrum of their diverse roles is yet to be understood (3).

The cancer-predisposing Lynch syndrome (LS) is an autosomal dominant disorder caused by germline mutations in the MMR pathway genes, mainly mutL homolog 1 (*MLH1*), mutS homolog 2 (*MSH2*), mutS homolog 6 (*MSH6*), epithelial cell adhesion molecule (*EPCAM*), and post-meiotic segregation increased 2 (*PMS2*) (3, 4). The LS can also result from mutations located in flanking regions of MMR genes (5, 6). Predominantly, the LS is caused by the presence of loss-of-function germline mutations in *MLH1* and *MSH2* genes (7), mutations in *MSH6* and *PMS2* are less frequent, and *EPCAM* is the less frequently mutated gene in the LS. The individuals with the LS tend to exhibit nucleotide loss or gain within the DNA microsatellite loci (microsatellite instability, MSI) (8), and their cells have a “mutator phenotype” which is causative to many types of malignancies.

The LS, originally identified as a disorder associated with colorectal cancer and previously known as hereditary non-polyposis colorectal cancer, is currently defined as a multi-tumor syndrome. The LS is found to be related to plethora of extracolonic malignancies including cancers of urinary tract, endometrial, small bowel and others (9).

Whether LS-associated malignancies include both ovarian cancer (OC) and breast cancer (BC) is yet an open topic of discussion. The link between the LS-associated germline mutations and hereditary OC has been demonstrated in several studies, and it is estimated that 10–15% of hereditary OC are LS-related (10). Recently, germline mutations in *MSH2, MSH6*, and *PMS2* genes have been found associated with BC (11, 12). However, neither the revised Amsterdam criteria for LS diagnosis nor the revised Bethesda criteria for MSI tests include BC (13), despite the data suggesting the link between BC and LS. In the recent publication “Lynch syndrome: five unanswered questions” the authors suggest that whether BC should be included or excluded from LS-related tumors is a perhaps the most important question in a field of “LS tumor spectrum” (14).

BC has a strong hereditary component and in many cases is caused by germline mutations in the predisposition genes such as DNA damage recognition and repair (DDR) genes *BRCA1, BRCA2*, and others, which are currently included into the multi-gene panels for BC risk assessment (15, 16). Nevertheless, a sizeable proportion of the patients with familial history of BC do not carry germline mutations in the currently known genes. Although some of such cases might be explained in part by the presence of heritable epigenetic marks (“epimutations”) leading to the disease (17), it's possible that germline pathogenic predisposing mutations in other, yet unknown genes exist, but are not identified yet (the “missing heritability” phenomena) (18). If BC is a part of LS, then pathogenic mutations in MMR pathway genes associated with BC should be included into the clinical genetic testing panels.

The Next Generation Sequencing (NGS) technologies are instrumental tools in molecular diagnostic allowing rapid and simultaneous analysis of broad panels of disease-associated germline mutations within multiple genes. The results of NGS-based tests for genetic risk assessment are concordant with conventional diagnostic methods, as demonstrated for hereditary BC and/or OC (19). Based on our pilot study indicating the presence of germline mutations within the MMR genes in patients with BC and familial history of cancer (20), we proposed that BC is a part of LS. To test this, we performed Targeted NGS of *MLH1, MSH2, MSH6, EPCAM*, and *PMS2* genes in a big cohort comprising of 711 patients with hereditary BC, 60 patients with sporadic cancer, and 492 healthy donors from Volga and Central Federal Districts, Russian Federation.

Our study demonstrates that the frequencies of the most of causative LS germline mutations are higher in patients with hereditary BC compared to healthy population control, and finds the association of several germline mutations within the genes *MLH1, MSH2, MSH6*, and *PMS2* with hereditary BC. This finding provides insights into the biology of LS and BC and supports including LS-associated mutations in genetic tests for the patients with hereditary BC in our study population.

## Materials and Methods

### Study Population

The study included 711 participants with hereditary BC and 60 participants with sporadic BC receiving a treatment for BC (chemotherapy and/or surgical treatment) at several medical centers in Volga Federal District, Russian Federation. The control comprised 492 healthy donors from Volga and Central Federal Districts, Russian Federation. The study participants self-identified with Slavic, Tatar or Bashkir ethnicities, and we ensured that both case and control group similarly reflect the ethnic diversity of the population from this geographical region. The criteria for inclusion into the patient's cohort, based on age of cancer manifestation, familial history of cancer, and clinical-pathological characteristics of the disease were previously described in our smaller scale pilot study (20). In particular, patients were included into the hereditary BC group if they had (1) BC diagnosis and familial history of any cancer (including kidney cancer, esophageal cancer, stomach cancer, lung cancer, sarcoma, colon cancer, leukemia, breast cancer, and ovarian cancer) in the first-, or second-, or third-degree relative or (2) BC manifestation at early age (before 30 y.o.) or (3) manifestation of triple negative BC at early age (before 35 y.o.). The estrogen receptor (ER), progesterone receptor (PR), human epidermal growth factor receptor 2 (HER2) and Ki67 status of the patients was established by clinical pathologist**s** as part of the routine patient care. The clinical and demographical characteristics for the patients are shown in Supplementary Table 1. All study participants provided informed consent prior enrolling to the study in accordance with Declaration of Helsinki.

### Targeted Next-Generation Sequencing

Genomic DNA was isolated from the whole blood samples using DNA Blood Mini QIAcube Kit (Qiagen), and 100 ng of DNA was used to generate Targeted NGS libraries. The target enrichment, sequencing and analysis were performed as described previously (20, 21) with slight modifications. In particular, KAPA HyperPlus (Roche) was used for library preparation and DNA enzymatic fragmentation, DNA was fragmented to the size of 150–200 b.p. Concentration of the DNA in the library was measured by Qubit (ThermoFisher Scientific) following manufacturer's instructions, the presence of the primer dimers was assessed using Agilent High Sensitivity DNA Kit (Agilent), the optimal length of the fragment including adapter was 290–330 b.p. Next, libraries were combined and hybridized with SeqCap EZ Choice (Roche), following manufacturer's recommendations. Hybridization was performed at +47°C for 16 h. SeqCap Capture beads were used for enrichment, and amplification was performed using KAPA HiFi HS MasterMix (Roche). Sequencing was performed using MiSeq (Illumina). The gene panel included *MLH1, MSH2, MSH6, EPCAM*, and *PMS2* genes (MMR pathway genes). In the study participants carrying mutations in the MMR pathway mutational status of the other genes associated with BC, namely namely *ATM, BRCA1, BRCA2, APC*, and *TP53* genes (DDR pathway genes), was also determined by NGS. Patients carrying pathogenic mutations in DDR pathway genes were excluded from analysis. *In-silico* tools SIFT, PolyPhen2, MutationTaster, CADD, DANN, M-CAP, and REVEL were used for the prediction of pathogenicity. All sequencing data were submitted to SRA database and can be accessed at https://www.ncbi.nlm.nih.gov/sra/PRJNA588789.

### Statistical Analysis

The data was analyzed using standard statistical tests as described previously (21). In particular, a two-tailed Fisher exact test was performed using the R software v.3.3 (*fisher.test* function). Statistical as significance was defined a *p* < 0.05.

## Results and Discussion

While the role of LS in hereditary OC is established and widely accepted, it is a long-standing question whether BC should also be classified under an umbrella of LS (22), as results of previous studies are inconsistent and contradictory. This lack of consistency might be explained by the inter-population and inter-ethnic differences and result from the unique ethnic-specific genetic traits within the study cohorts (23). It is apparent now that the epidemiology and distribution of pathogenic germline mutations in BC are population-specific (24, 25). Thus, the population- and ethnic background of the patient should be considered at the stage of the cancer risk genetic evaluation (23, 26), as genetic risks might be mis-estimated if based on a data obtained in a study population with different ancestral (and, thus, genetic) background. Moreover, genetic studies of the complex hereditary diseases in understudied populations provide a unique opportunity to identify novel genetic markers. Currently, a large body of data on genetics of familial BC exists for some well-studied populations and ethnic groups, while some populations and ethnicities remain understudied, resulting in a so-called “social inequity in cancer.” Hence, further studies focusing on ethnic-specific and population-specific aspects of hereditary BC are of definite clinical value.

Previously, we demonstrated that the spectrum and frequencies of pathogenic nucleotide variants in DDR pathway genes in Tatar patients with familial OC and/or BC from Kazan region of the Volga Federal District of the Russian Federation are different from ones reported for the patients of Slavic descent from Moscow (21). Here, we applied Targeted NGS to determine the prevalence and spectrum of germline mutations in MMR pathway genes in the patients with hereditary BC from the Volga and Central Federal Districts of the Russian Federation and in healthy donors of the similar ethnical backgrounds. We performed Targeted NGS and, in a cohort comprising 711 participants with hereditary BC, identified in 17 participants 10 mutations in *MLH1* gene (c.945C>G, c.1637A>G, c.803A>G, c.1321G>A, c.1937A>G, c.-7C>T, c.2194A>G, c.472A>G, c.2194A>G, andc.1090A>C), in 9 participants 5 mutations in *MSH2* gene (c.260C>G, c.2178G>C, c.2178G>C, c.2197G>A, c.815C>T), in 19 participants 19 mutations in *MSH6* gene (c.4004A>C, c.2291C>A, c.2156C>T, c.2673C>G, c.893G>A, c.2554_2556del, c.3674C>T, c.3674C>T, c.3986C>T, c.3674C>T, c.2503C>G, c.3217C>T, c.3254dupC, c.3259C>T, c.1063G>A, c.3951T>G, c.968C>G, c.1481C>T, c.3151G>A), in 6 participants 3 mutations in *EPCAM* gene (c.557A>C, c.859-3C>G, c.272A>T), and in 20 participants 14 mutations in *PMS2* gene (c.1642G>A, c.1268C>G, c.1268C>G, c.86G>C, c.944G>A, c.1567T>A, c.2438G>A, c.1399G>A, c.2149G>A, c.1630G>A,c.1753C>T, c.1595A>G, c.1630G>A, c.1901A>G). In a group of 60 patients with sporadic BC we found no germline mutations in MMR pathway genes. In a group of 462 healthy participants, one germline mutation in *EPCAM* gene (c.859-3C>G) was found in 2 participants. All mutations were heterozygous. The mutations included variants of unknown/uncertain significance (VUS), Likely pathogenic and Pathogenic mutations, based on The Human Mutations Database (HGMD) and the ClinVar database.

**Figure 1:**
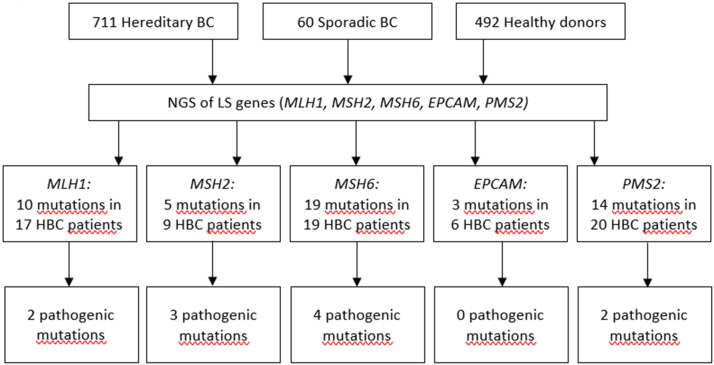
Pathogenic mutations detected in LS genes.

The presence of the mutations in the DDR pathway genes was also assessed in the study participants currying mutations in the MMR pathway. Among the patients with mutations in MMR pathway, 17 (2.4%) also harbored pathogenic mutations in the DDR pathway genes (Supplementary Table 2). If such mutations in DDR were present, the OR for mutation in MMR pathway genes was calculated twice, including and excluding cases with mutations in DDR. Only OR calculated for cases with germline mutations in MMR pathway genes and without pathogenic germline mutations in DDR genes was used to assess the pathogenicity of the mutation. Some of mutations were detected more than once in the patient cohort, the most recurrent ones were c.1321G>A in *MLH1*, c.260C>G in *MSH2*, and c.86G>C in *PMS2* genes. The spectrum and frequencies of the mutations in the study cohort are shown in Supplementary Table 2. Sixty-nine patients (9.7%) harbored at least one germline mutation in the MMR pathway genes, of them thirty-two patients harbored mutations in MMR pathway genes which we define as pathogenic or likely pathogenic, and of them twenty-six patients (3.6%) did not have any pathogenic mutations in DDR pathway genes, compared to two mutations in MMR pathway genes (0.4%) detected in a group of 492 healthy donors [*p* = 0.00013, *OR* = 8.9 (CI 95% 2.2–78.4] (Figure 1, Supplementary Tables 2, 3). The age of occurrence of the clinical manifestations of disease in hereditary BC patients with and without LS-mutations was 45.3 ± 9.7 and 47.1 ± 11.3 years, respectively, compared to 58.9 ± 9.3 in a group of patients with sporadic BC. The percentage of HER2+ patients in hereditary BC patients with and without LS-mutations was 34 and 30%, respectively.

We detected 10 different mutations in *MLH1, 5* in *MSH2, 19* in *MSH6, 3* in *EPCAM*, and 14 in *PMS2* genes in a cohort of participants with hereditary BC, while only one of them was found in the reference healthy control (Figure 1, Supplementary Table 2). In the samples from the Exome Aggregation Consortium database (http://gnomad.broadinstitute.org/) the frequencies of these mutations are extremely low and similar to the data obtained in our study population in the control group (Supplementary Table 2). In other populations, the frequency of *MSH6* gene mutations was determined as 0.2% in a study performed in Germany in a group of patients with BC and/or OC (27), and in a study performed in USA in a group of females with stages I to III of BC (28). The rate of Ashkenazi Jewish founder mutations c.3984_3987dupGTCA and c.3959_3962delCAAG within *MSH6* gene in a study population comprising 1016 participants with familial history of BC and/or OC was 0.49% (29). Additionally, the recent case report suggests an association between sporadic BC and biallelic mutations in *MSH6* (30). In a small cohort of triple-negative BC patients with early onset or familial history of cancer the frequency of *MSH2* gene mutations was 4% (31). The cohort study of several families in UK suggested germline mutation carriers in MLH1 gene are at moderate risk of BC (32). Recently, study by Roberts et al. reported that *MSH6* and *PMS2* germline pathogenic variants are associated with increased risk of BC (12). However, this association was not confirmed in the other study (33). It was suggested that the ascertainment bias might has affected the interpretation of the data by Roberts et al., as the study cohort was enriched with participants with BC diagnosis, and the BC risk in the total study cohort doubled BC risk in the reference cohort (general population) (33). Other studies found either no association of *PMS2* with BC (34), or demonstrated that carriers of mutations in *PMS2* gene had significant standardized incidence ratios for OC and BC (35).

The results of our study are in line with the previous studies demonstrating an association of germline mutations in *MLH1, MSH2, MSH6*, and *PMS2* genes with hereditary BC. There was also a tendency for the carriers of LS-mutations to have earlier manifestation of the disease (45.3 ± 9.7 y.o. with LS-mutations and 47.1 ± 11.3 y.o. without). However, given the importance of clinical decisions in BC risk assessment, we agree with Evans et al. (34) that the decision of the genetic counseling specialist should be based “on the overall evidence available.”

We also suggest changing the classification of several mutations characterized in this study, based on variant interpretation standards and guidelines of the American College of Medical Genetics and Genomics and the Association for Molecular Pathology. In particular, currently the c.1321G>A in *MLH1* gene is defined in databases as VUS, but we suggest that it is a Likely pathogenic mutation [*OR* = 10.9 (3.4–26.6), *p* = 0.0001, Supplementary Table 2]. The 260C>G in *MSH2* gene is also VUS, but we suggest it should be classified as a Pathogenic mutation [*OR* = 361.4 (51.9–4387.9), *p* = 1.359e-08, Supplementary Table 2]. The c.2178G>C in *MSH2* is classified as VUS, but based on our study we suggest that it is a Likely pathogenic mutation [*OR* = 53.4 (5.2–297.8), *p* = 0.001, Supplementary Table 2]. The c.3217C>T in *MSH6* gene is classified as VUS, but we suggest it is a Likely pathogenic mutation [*OR* = 19.5 (3.8–63.2), *p* = 0.0007, Supplementary Table 2]. Finally, the c.859-3C>G in *EPCAM* gene is classified as VUS, but we propose that it is a Likely Benign mutation, based on its relatively high frequency in our healthy control group (much higher than in the gnomAD).

The large number of the mutations within the *MSH6* gene among the patients in our study cohort is of particular interest. The currently available MSI tests based on mononucleotide repeat markers BAT25, BAT26, NR21, NR24, and MONO27 often give false-negative results in case of *MSH6* gene (36), thus other methods of MSI detection, such as IHC, are more sensitive. Given that *MSH6* is the most frequently mutated LS gene in BC in our study population, we suggest using IHC as a preferential method of MSI assessment in hereditary BC.

There are several limitations of our study. Firstly, the populations of the Volga and Central Federal District comprise several ethnicities, thus differences in mutation frequencies between control and case group might be attributed to specific genetic traits of the participants of different descent, rather than biology of LS and BC. This highlights an importance of ensuring that ethnic groups are equally represented in both case and control groups. We tried to address this by recruiting a large number of participants and ensuring that both case and control group similarly reflect the ethnic diversity of the population. Secondly, some patients with germline mutations in MMR pathway genes may still have a tumor with functional MMR proteins, as previously described (37). This advocates for more rigorous testing, especially when choosing tailored therapy. In future studies, this should be addressed by applying Targeted NGS for analysis of both tumor biopsy and whole blood tissue samples, which was beyond the scope of the current study.

The fundamental step in cancer risk evaluation, prevention and clinical surveillance of hereditary cancers is a detection of predisposing germline mutations in individuals with familial history of cancer. In many cases the therapy decisions are also guided by the genotype of a patient (the clinical approach known as personalized therapy) as carriers of different allelic variants may respond to the treatment differently. For example, *MSH2*-deficient cancer cells are selectively sensitive to Methotrexate, and it's been proposed that patients with *MSH2* deficiency will respond to the Methotrexate therapy (38). Another compound selectively targeting MMR-deficient cancer cells is FDA approved drug Triamterene (39). It has also been shown that patients with high MSI and mutations in MMR genes have favorable response to the PD-1 blockade immunotherapy in a broad spectrum of cancers (40). Finally, several mutations in MMR pathway genes have been found associated with radiosensitivity in BC patients (41). Thus, determining mutation status of the MMR pathway genes can guide personalized therapy. Additionally, the carriers of LS-related germline mutations identified by the genetic tests benefit from the chemoprevention therapies (42), that would not otherwise be subscribed in the absence of a suggestive clinical evidence and prior to the manifestation of the disease.

The pathogenic mutations in genes from MMR pathway result in compromised DNA repair. The defects in DNA repair are associated with increased neoantigen load and linked to the elevated expression of immunosuppressive PD-L1 by the cancer cells (43). Patients with tumors expressing high level of PD-L1 benefit from the immune checkpoint blockade therapy, thus identification of such patients has important clinical implications. The correlation between the level of PD-L1 and both methylation of *BRCA1* gene and its mutation status has been found in OC (44, 45). We propose that in hereditary BC the PD-L1 level may correlate with the presence of pathogenic mutations in the genes from both MMR and DDR pathways (such as *BRCA1/2* and others), and suggest that in future studies such correlation should be assessed as a potential clinical biomarker.

In conclusion, our study demonstrates the relatively frequent presence of the germline LS-mutations in the patients with hereditary BC, and association of hereditary BC with c.1321G>A in *MLH1*, c.260C>G and c.2178G>C in *MSH2*, c.3217C>T in *MSH6*, and c.1268C>G and c.86G>C in *PMS2* genes. We recommend including MMR pathway genes into the multi-gene panels for risk assessment of hereditary BC, based on the overall clinical picture.

## Data Availability Statement

All sequencing data were submitted to SRA database and can be accessed at https://www.ncbi.nlm.nih.gov/sra/PRJNA588789.

## Ethics Statement

This studies involving human participants were reviewed and approved by the study was carried out in accordance with the recommendations of International code of medical ethics and local ethical committees of Kazan Federal University and Tatarstan Cancer Center. In accordance with the Declaration of Helsinki all study participants gave written informed consent to participate in this study.

## Author Contributions

AN, DS, ES, LS, MG, MD, OB, and RE: data collection, analysis, and interpretation. AN, DC, ES, MG, and OG: study conception and design. AN, DC, OB, and OG: drafting and critical revision of the manuscript.

## Conflict of Interest

MD was employed by Druzhkov Clinic Ltd., Russia. MG was employed by National Bioservice LLC, Russia. The remaining authors declare that the research was conducted in the absence of any commercial or financial relationships that could be construed as a potential conflict of interest.
