# Supplementary material for: Lynch Syndrome Germline Mutations in Breast Cancer: Next Generation Sequencing Case-Control Study of 1,263 Participants
Source: Front Oncol. 2020 May 29;10:666. doi: 10.3389/fonc.2020.00666 (PMC7273971; doi:10.3389/fonc.2020.00666)
Supplement: Supplementary file 1 [file Data_Sheet_1.docx]

**Supplementary Table 1.** **Clinical and demographic characteristics of the study cohort**

| **Age** | **Age of disease manifestation** | **HER2** | **ER** | **PR** |
| --- | --- | --- | --- | --- |
| Hereditary BC (N = 711) | | | | |
| 49.4 ± 11.6 | 47.1 ± 11.3 | Triple-negative (N = 275, 39%)  HER2-positive (N = 215, 30%)  ER+, PR+, HER2- (N = 172, 24%) | | |
| Hereditary BC with LS mutations (32 from 711) | | | | |
| 48.4 ± 10.4 | 45.3 ± 9.7 | Triple-negative (N = 7, 22%)  HER2-positive (N = 11, 34%)  ER+, PR+, HER2- (N = 8, 25%) | | |
| Sporadic BC (N = 60) | | | | |
| 60.7 ± 9.1 | 58.9 ± 9.3 | Triple-negative (N = 10, 16%)  HER2-positive (N = 22, 37%)  ER+, PR+, HER2- (N = 27%) | | |
| Control (N = 492) | | | | |
| 55.2 ± 12.9 | - | - | - | - |

Values calculated as mean ± SD

**Supplementary Table 2. The spectrum and frequencies of the mutations in participants with hereditary BC vs healthy control**

| **Patient N** | hg19 | cDNA | Protein | mutation type | localisation | gnomAD, max frequency in exomes | dbSNP | | Pathogenity from HGMD/ClinVar | other pathogenic mutations | Frequency in BC  (711) | Frequency in healthy control (492) | OR, CI 95% (max frequency from еNFE/еEAS) |
| --- | --- | --- | --- | --- | --- | --- | --- | --- | --- | --- | --- | --- | --- |
| **MLH1 (NM_000249.3)** | | | | | | | | | | | | | |
| P1 | 3:37061861 | c.945C>G | p.H315Q | Missense | exon | NFE - 0.00004 | rs587779959 | | VUS | - | 0.0014 (1) | 0 | 36.3 (0.8 -322.8)  *p* = 0.032 |
| P2 | 3:37081755 | c.1637A>G | p.K546R | Missense | exon | NFE - 0.00002 | rs587779954 | | VUS | - | 0.0014 (1) | 0 | 79.9 (1.4 - 1617.3)  *p* = 0.019 |
| P3 | 3:37059009 | c.803A>G | p.E268G | Missense | exon | NFE - 0.0004 | rs63750650 | | Likely pathogenic  (29–31) | - | 0.0014 (1) | 0 | 7.9 (0.2 - 48.8)  *p* = 0.1 |
| P4 | 3:37067410 | c.1321G>A | p.A441T | Missense | exon | NFE - 0.0006 | rs63750365 | | VUS | - | 0.0070 (5) | 0 | 10.9 (3.4 -26.6)  *p* = 0.0001 |
| P5 | 3:37067410 | c.1321G>A | p.A441T | Missense | exon |  |  |  |  | - |  |  |  |
| P6 | 3:37067410 | c.1321G>A | p.A441T | Missense | exon |  |  |  |  | - |  |  |  |
| P7 | 3:37067410 | c.1321G>A | p.A441T | Missense | exon |  |  |  |  | - |  |  |  |
| P8 | 3:37067410 | c.1321G>A | p.A441T | Missense | exon |  |  |  |  | - |  |  |  |
| P9 | 3:37090048 | c.1937A>G | p.Y646C | Missense | exon | AFR - 0.0001  NFE - 0.00008 | rs35045067 | | Likely pathogenic  (32–34) | BRCA1:NM_007294.3:c.5266dupC:p.Q1756fs | 0.0014 (1) | 0 | 20.2 (0.5 -145.7)  *p* = 0.05 |
| P10 | 3:37035032 | c.-7C>T | - | - | 5'UTR | NFE - 0.0004 | rs104894994 | | VUS | - | 0.0042 (3) | 0 | 4.4 (0.9 - 13.1)  *p* = 0.03 |
| P11 | 3:37035032 | c.-7C>T | - | - | 5'UTR |  |  |  |  | - |  |  |  |
| P12 | 3:37035032 | c.-7C>T | - | - | 5'UTR |  |  |  |  | - |  |  |  |
| P33 | 3:37092067 | c.2194A>G | p.K732E | Missense | exon | - | - | | VUS | BRCA1:NM_007294.3:c.3772delG:p.E1258fs | 0.0028 (2) | 0 | NA |
| P34 | 3:37050323 | c.472A>G | p.N158D | Missense | exon | - | - | | VUS | - | 0.0014 (1) | 0 | NA |
| P35 | 3:37092067 | c.2194A>G | p.K732E | Missense | exon | - | - | | VUS | - | 0.0028 (2) | 0 | NA |
| P36 | 3:37067179 | c.1090A>C | p.T364P | missense | exon | - | - | | VUS | - | 0.0014 (1) | 0 | NA |
| P37 | 3:37092067 | c.2194A>G | p.K732E | missense | exon | - | - | | VUS | - | 0.0028 (2) | 0 | NA |
| **MSH2 (NM_000251.2)** | | | | | | | | | | |  |  |  |
| P38 | 2:47635588 | c.260C>G | p.S87C | missense | exon | NFE - 0.000015 | rs587781447 | | VUS | - | 0.0070 (5) | 0 | 448.1 (74.1 - 5429.5)  *p* = 1.057e-10  without BRCA2 p.K434fs:  361.4 (51.9 - 4387.9)  *p* = 1.359e-08 |
| P39 | 2:47635588 | c.260C>G | p.S87C | missense | exon |  |  |  |  | - |  |  |  |
| P40 | 2:47635588 | c.260C>G | p.S87C | missense | exon |  |  |  |  | - |  |  |  |
| P41 | 2:47635588 | c.260C>G | p.S87C | missense | Exon |  |  |  |  | BRCA2:NM_000059.3:c.1301_1304del:p.K434fs |  |  |  |
| P42 | 2:47635588 | c.260C>G | p.S87C | missense | Exon |  |  |  |  | - |  |  |  |
| P43 | 2:47703678 | c.2178G>C | p.M726I | missense | exon | NFE - 0.00005 | rs587782396 | | VUS | - | 0.0028 (2) | 0 | 53.4 (5.2 - 297.8)  *p* = 0.001 |
| P44 | 2:47703678 | c.2178G>C | p.M726I | missense | exon |  |  |  |  | - |  |  |  |
| P50 | 2:47703697 | c.2197G>A | p.A733T | missense | exon | EAS - 0.0003  NFE – 0 | rs772662439 | | VUS | - | 0.0014 (1) | 0 | 4.3 (0.09 - 35.6)  *p* = 0.2 |
| P51 | 2:47641430 | c.815C>T | p.A272V | missense | exon | NFE - 0.0003 | rs34136999 | | Likely pathogenic  (35–40) | - | 0.0014 (1) | 0 | 4.3 (0.09 - 20.7)  *p* = 0.2 |
| **MSH6 (NM_000179.2)** | | | | | | | | | | | | | |
| P61 | 2:48033920 | c.4004A>C | p.E1335A | missense | exon | AMR - 0.0002  NFE - 0.00004 | rs564434147 | | Likely pathogenic (41,42) | APC:NM_000038.5:c.6497G>A:p.R2166Q | 0.0028 (2) | 0 | 70.4(6.7 - 429.7)  *p* = 0.0007  without APCp.R2166Q or BRCA1p.Q1756fs:  35.2 (0.7 - 313.1)  *p* = 0.03 |
| P62 | 2:48033920 | c.4004A>C | p.E1335A | missense | exon |  |  |  |  | BRCA1:NM_007294.3:c.5266dupC:p.Q1756fs |  |  |  |
| P63 | 2:48027413 | c.2291C>A | p.T764N | missense | exon | AFR - 0.0001  NFE - 0.000007 | rs561198849 | | Likely pathogenic (43–45) | BRCA2:NM_000059.3:c.2897_2898del:p.T966fs | 0.0014 (1) | 0 | 180.4 (2.3 - 12299.6)  *p* = 0.01 |
| P2 | 2:48027278 | c.2156C>T | p.T719I | missense | exon | NFE - 0.00002 | rs373418713 | | VUS | - | 0.0014 (1) | 0 | 90.4(1.5 - 1751.2)  *p* = 0.01 |
| P65 | 2:48027795 | c.2673C>G | p.I891M | missense | exon | AFR - 0.00006  NFE - 0.000009 | rs146006741 | | VUS | BRCA1:NM_007294.3:c.5266dupC:p.Q1756fs | 0.0014 (1) | 0 | 158.8 (2.0 - 10986.6)  *p* = 0.01 |
| P69 | 2:48026015 | c.893G>A | p.R298Q | missense | exon | NFE - 0.000026 | rs765237563 | | VUS | - | 0.0014 (1) | 0 | 53.3 (1.01 - 660.4)  *p* = 0.02 |
| P72 | 2:48027675 | c.2554_2556del | p.852_852del | in-frame del | exon | NFE - 0.00002 | rs587782858 | | Pathogenic  (46–49) | - | 0.0014 (1) | 0 | 39.6 (0.8 - 396.7)  *p* = 0.03 |
| P73 | 2:48033370 | c.3674C>T | p.T1225M | missense | exon | NFE - 0.0002 | rs63750370 | | VUS | - | 0.0028 (2) | 0 | 10.7 (0.3 - 68.3)  *p* = 0.09 |
| P74 | 2:48033775 | c.3986C>T | p.S1329L | missense | exon | NFE - 0.0001 | rs199594809 | | VUS | BRCA1:NM_007294.3:c.5266dupC:p.Q1756fs | 0.0014 (1) | 0 | 13.7 (0.3 - 91.5)  *p* = 0.07 |
| P75 | 2:48033370 | c.3674C>T | p.T1225M | missense | exon | NFE - 0.0002 | rs63750370 | | VUS | - | 0.0028 (2) | 0 | 10.7 (0.3 - 68.3)  *p* = 0.09 |
| P6 | 2:48027625 | c.2503C>G | p.Q835E | missense | exon | EAS - 0.00005  NFE– 0 | rs63751321 | | VUS | - | 0.0014 (1) | 0 | 25.7 (0.3 - 1983.4)  *p* = 0.07 |
| P77 | 2:48030603 | c.3217C>T | p.P1073S | missense | exon | NFE - 0.0002 | rs142254875 | | VUS | - | 0.0042 (3) | 0 | 19.5 (3.8 - 63.2)  *p* = 0.0007 |
| P78 | 2:48030603 | c.3217C>T | p.P1073S | missense | exon |  |  |  |  | - |  |  |  |
| P79 | 2:48030603 | c.3217C>T | p.P1073S | missense | exon |  |  |  |  | - |  |  |  |
| P80 | 2:48030639 | c.3254dupC | p.T1085fs | frameshiftins | exon | NFE - 0.00006 | rs267608087 | | Pathogenic  (50–57) | - | 0.0014 (1) | 0 | 22.5 (0.5-168.2)  *p* = 0.04 |
| P73 | 2:48030645 | c.3259C>T | p.P1087S | missense | exon | NFE - 0.0002 | rs63750998 | | VUS | - | 0.0014 (1) | 0 | 5.7(0.1-34.1)  *p* = 0.2 |
| P106 | 2:48026185 | c.1063G>A | p.G355S | missense | exon | EAS - 0.0025  NFE – 0 | rs587778531 | | VUS | - | 0.0014 (1) | 0 | 0.9  *p* = 1 |
| P107 | 2:48033740 | c.3951T>G | p.H1317Q | missense | exon | AFR - 0.00008  NFE – 0 | rs764786814 | | VUS | - | 0.0014 (1) | 0 | NA |
| P109 | 2:48026090 | c.968C>G | p.T323S | missense | exon | - | - | | VUS | TP53:NM_000546.5:c.523C>T:p.R175C | 0.0014 (1) | 0 | NA |
| P109 | 2:48026603 | c.1481C>T | p.A494V | missense | exon | - | - | | VUS |  | 0.0014 (1) | 0 | NA |
| P111 | 2:48028273 | c.3151G>A | p.V1051I | missense | exon | SAS - 0.007  NFE – 0 | rs576269342 | | VUS | - | 0.0014 (1) | 0 | NA |
| **EPCAM (NM_002354.2)** | | | | | | | | | | | | | |
| P112 | 2:47606093 | c.557A>C | p.Y186S | missense | exon | NFE - 0.00002 | rs757739862 | | VUS | - | 0.0014 (1) | 0 | 60.3 (1.1 -738.3)  *p* = 0.02 |
| P113 | 2:47612302 | c.859-3C>G | - | splicing | splice site | NFE - 0.0001 | rs201314303 | | VUS | - | 0.0042 (3) | 0.0041 (2) | 41.9 (7.6 -153.1)  *p* = 8.785e-05 |
| P114 | 2:47612302 | c.859-3C>G | - | splicing | splice site |  |  |  |  | - |  |  |  |
| P115 | 2:47612302 | c.859-3C>G | - | splicing | splice site |  |  |  |  | - |  |  |  |
| P122 | 2:47601034 | c.272A>T | p.N91I | missense | exon | - | - | | VUS | - | 0.0028 (2) | 0 | NA |
| P123 | 2:47601034 | c.272A>G | p.N91S | missense | exon | - | - | | VUS | ATM:NM_000051.3:c.8147T>C:p.V2716A |  |  |  |
| **PMS2 (NM_000535.6)** | | | | | | | | | | | | | |
| P124 | 7:6026754 | c.1642G>A | p.D548N | missense | exon | NFE - 0.00002 | rs374591423 | VUS | | BRCA1:NM_007294.3:c.5266dupC:p.Q1756fs | 0.0014 (1) | 0 | 79.8 (1.4 -1615.3)  *p* = 0.02 |
| P125 | 7:6027128 | c.1268C>G | p.A423G | missense | exon | NFE - 0.0001 | rs756883400 | VUS | | - | 0.0028 (2) | 0 | 20.1 (2.3 -84.5)  *p* = 0.005 |
| P126 | 7:6027128 | c.1268C>G | p.A423G | missense | exon |  |  |  |  | - |  |  |  |
| P127 | 7:6045600 | c.86G>C | p.G29A | missense | exon | NFE - 0.0005 | rs146176004 | VUS | | - | 0.0098 (7) | 0 | 18.4 (7.1-40.3)  *p* = 2.427e-07  without BRCA1 p.Q1756fs:  15.8 (5.6- 36.4)  *p* = 4.031e-06 |
| P128 | 7:6045600 | c.86G>C | p.G29A | missense | Exon |  |  |  |  | - |  |  |  |
| P129 | 7:6045600 | c.86G>C | p.G29A | missense | Exon |  |  |  |  | - |  |  |  |
| P130 | 7:6045600 | c.86G>C | p.G29A | missense | Exon |  |  |  |  | - |  |  |  |
| P131 | 7:6045600 | c.86G>C | p.G29A | missense | Exon |  |  |  |  | - |  |  |  |
| P132 | 7:6045600 | c.86G>C | p.G29A | missense | Exon |  |  |  |  | BRCA1:NM_007294.3:c.5266dupC:p.Q1756fs |  |  |  |
| P133 | 7:6045600 | c.86G>C | p.G29A | missense | Exon |  |  |  |  | - |  |  |  |
| P62 | 7:6031648 | c.944G>A | p.R315Q | missense | exon | - | rs116314131 | VUS | | BRCA1:NM_007294.3:c.5266dupC:p.Q1756fs | 0.0014 (1) | 0 | NA |
| P166 | 7:6026829 | c.1567T>A | p.S523T | missense | exon | NFE - 0.0002 | rs63751132 | VUS | | - | 0.0014 (1) | 0 | 6.5(0.2 -39.3)  *p* = 0.1 |
| P167 | 7:6017226 | c.2438G>A | p.R813Q | missense | exon | EAS - 0.0002  NFE – 0 | rs587782665 | VUS | | - | 0.0014 (1) | 0 | 7.3 (0.1 -91.7)  *p* = 0.2 |
| P168 | 7:6026997 | c.1399G>A | p.V467I | missense | exon | AFR - 0.0001  NFE - 0.00005 | rs373611083 | VUS | | - | 0.0014 (1) | 0 | 25.9 (0.6 -203.2)  *p* = 0.04 |
| P170 | 7:6022480 | c.2149G>A | p.V717M | missense | exon | NFE - 0.0006 | rs201671325 | VUS | | - | 0.0014 (1) | 0 | 2.06 (0.05 -11.8)  *p* = 0.4 |
| P171 | 7:6026766 | c.1630G>A | p.D544N | missense | exon | - | rs876660139 | VUS | | BRCA1:NM_007294.3:c.5251C>T:p.R1751X | 0.0028 (2) | 0 | NA |
| P172 | 7:6026643 | c.1753C>T | p.L585F | missense | exon | - | - | VUS | | BRCA1:NM_007294.3:c.181T>G:p.C61G | 0.0014 (1) | 0 | NA |
| P174 | 7:6026801 | c.1595A>G | p.H532R | missense | exon | - | - | VUS | | - | 0.0014 (1) | 0 | NA |
| P175 | 7:6026766 | c.1630G>A | p.D544N | missense | exon | - | rs876660139 | VUS | | BRCA1:NM_007294.3:c.5251C>T:p.R1751X | 0.0028 (2) | 0 | NA |
| P176 | 7:6026495 | c.1901A>G | p.H634R | missense | exon | - | rs767904893 | VUS | | BRCA1:NM_007294.3:c.5266dupC:p.Q1756fs | 0.0014 (1) | 0 | NA |

**Supplementary Table 3. Clinical and demographic characteristics of all pathogenic mutations' carriers**

| **Patient N** | hg19  cDNA  Protein | Age | Age of manifestation | ER | PR | HER2 | Ki-67 | BC family history | Cancer family history | Pathogenity from our study (by ACMG) |
| --- | --- | --- | --- | --- | --- | --- | --- | --- | --- | --- |
| **MLH1** | 42.6 ± 7.8 | 40.4 ± 8.5 |  | | | | | | | |
| P3 | 3:37059009  c.803A>G  p.E268G | 51 | 51 | 0 | 0 | 0 | 35% | None | Yes | Likely pathogenic |
| P4 | 3:37067410  c.1321G>A  p.A441T | 33 | 33 | 1+ | 0 | 3+ | 30% | None | Yes | Likely pathogenic |
| P5 |  | 50 | 50 | 4 | 3 | 1+ | 15% | Mother | Yes |  |
| P6 |  | 33 | 30 | 7 | 8 | 3+ | 10% | None | Yes |  |
| P7 |  | 36 | 35 | 6 | 0 | 0 | 60% | None | Yes |  |
| P8 |  | 47 | 46 | 0 | 0 | 3+ | 40% | None | Yes |  |
| P9 | 3:37090048  c.1937A>G  p.Y646C | 38 | 38 | - | - | - | - | None | Yes | VUS |
|  | | | | | | | | | | |
| **MSH2** | 50.8 ± 11.1 | 48.0 ± 9.7 |  | | | | | | | |
| P38 | 2:47635588  c.260C>G  p.S87C | 32 | 32 | - | - | - | - | Aunt | Yes | Pathogenic |
| P39 |  | 48 | 44 | 7 | 4 | 3+ | 40% | None | Yes |  |
| P40 |  | - | - | - | - | - | - | None | Yes |  |
| P41 |  | 56 | 49 | 0 | 0 | 0 | 50% | Aunt | Yes |  |
| P42 |  | 49 | 48 | 7 | 4 | 3+ | 40% | None | Yes |  |
| P43 | 2:47703678  c.2178G>C  p.M726I | 65 | 60 | 8 | 6 | 0 | 12% | Sister | Yes | Likely pathogenic |
| P44 |  | - | - | - | - | - | - | None | Yes |  |
| P51 | 2:47641430  c.815C>T  p.A272V | 55 | 55 | 8 | 3 | 0 | 40% | Sister | Yes | Likely pathogenic |
|  | | | | | | | | | | |
| **MSH6** | 52.0 ± 9.9 | 47.3 ± 11.3 |  | | | | | | | |
| P61 | 2:48033920  c.4004A>C  p.E1335A | 45 | 44 | 0 | 0 | 0 | 15% | None | Yes | VUS |
| P62 |  | 42 | 41 | 7 | 7 | 0 | 80% | Sister | Yes |  |
| P63 | 2:48027413  c.2291C>A  p.T764N | 62 | 61 | 0 | 0 | 0 | 40% | None | Yes | Likely pathogenic |
| P72 | 2:48027675  c.2554_2556del  p.852_852del | 56 | 55 | 0 | 0 | 3+ | 50% | None | Yes | Pathogenic |
| P77 | 2:48030603  c.3217C>T  p.P1073S | 44 | 43 | 0 | 0 | 0 | 65% | None | Yes | Likely pathogenic |
| P78 |  | 41 | 41 | 3+ | 3+ | 0 | 11% | None | Yes |  |
| P79 |  | 63 | 30, recurrence in 56 | 8 | 5 | 0 | 18% | Sister | Yes |  |
| P80 | 2:48030639  c.3254dupC  p.T1085fs | 41 | 41 | 0 | 0 | 2 | 30% | Mother | Yes | Pathogenic |
|  | | | | | | | | | | |
| **PMS2** | 53.3 ± 11.6 | 48.5 ± 10.9 |  | | | | | | | |
| P125 | 7:6027128  c.1268C>G  p.A423G | 60 | 59 | 8 | 8 | 0 | 8% | Sister | Yes | Likely pathogenic |
| P126 |  | 52 | 51 | 0 | 0 | 0 | 75% | Aunt | Yes |  |
| P127 | 7:6045600  c.86G>C  p.G29A | 51 | 47 | - | - | - | - | None | Yes | Likely pathogenic |
| P128 |  | 35 | 35 | 4 | 4 | 0 | 15% | None | Yes |  |
| P129 |  | - | - | 0 | 0 | 3+ | 70% | None | Yes |  |
| P130 |  | 62 | 52 | 3+ | 2+ | 2+ | - | None | Yes |  |
| P131 |  | 55 | 43 | 7 | 4 | 0 | 34% | Sister | Yes |  |
| P132 |  | 66 | 66 | 0 | 0 | 0 | 35% | None | Yes |  |
| P133 |  | 37 | 35 | 0 | 0 | 3+ | 25% | Grandmother | Yes |  |
